# Clinical Decision Support for Hypertension Management in Chronic Kidney Disease: A Randomized Clinical Trial

**DOI:** 10.1001/jamainternmed.2023.8315

**Published:** 2024-03-11

**Authors:** Lipika Samal, John L. Kilgallon, Stuart Lipsitz, Heather J. Baer, Allison McCoy, Michael Gannon, Sarah Noonan, Ryan Dunk, Sarah W. Chen, Weng Ian Chay, Richard Fay, Pamela M. Garabedian, Edward Wu, Matthew Wien, Saul Blecker, Hojjat Salmasian, Joseph V. Bonventre, Gearoid M. McMahon, David W. Bates, Sushrut S. Waikar, Jeffrey A. Linder, Adam Wright, Patricia Dykes

**Affiliations:** 1 Division of General Internal Medicine, Brigham and Women’s Hospital, Boston, Massachusetts; 2 Division of Renal Medicine, Brigham and Women’s Hospital, Boston, Massachusetts; 3 Harvard Medical School, Boston, Massachusetts; 4 Hackensack Meridian School of Medicine, Nutley, New Jersey; 5 Harvard T.H. Chan School of Public Health, Boston, Massachusetts; 6 Department of Biomedical Informatics, Vanderbilt University, Nashville, Tennessee; 7 Eastern Virginia Medical School, Norfolk; 8 USC School of Medicine Greenville, Greenville, South Carolina; 9 Mass General Brigham, Somerville, Massachusetts; 10 Alabama College of Osteopathic Medicine, Dothan; 11 Department of Medicine, NYU Grossman School of Medicine, New York, New York; 12 Children’s Hospital of Philadelphia, Philadelphia, Pennsylvania; 13 Section of Nephrology, Boston Medical Center, Boston University Chobanian & Avedisian School of Medicine, Boston, Massachusetts; 14 Division of General Internal Medicine, Northwestern University Feinberg School of Medicine, Chicago, Illinois

## Abstract

**Importance:**

Chronic kidney disease (CKD) affects 37 million adults in the United States, and for patients with CKD, hypertension is a key risk factor for adverse outcomes, such as kidney failure, cardiovascular events, and death.

**Objective:**

To evaluate a computerized clinical decision support (CDS) system for the management of uncontrolled hypertension in patients with CKD.

**Design, Setting, and Participants:**

This multiclinic, randomized clinical trial randomized primary care practitioners (PCPs) at a primary care network, including 15 hospital-based, ambulatory, and community health center–based clinics, through a stratified, matched-pair randomization approach February 2021 to February 2022. All adult patients with a visit to a PCP in the last 2 years were eligible and those with evidence of CKD and hypertension were included.

**Intervention:**

The intervention consisted of a CDS system based on behavioral economic principles and human-centered design methods that delivered tailored, evidence-based recommendations, including initiation or titration of renin-angiotensin-aldosterone system inhibitors. The patients in the control group received usual care from PCPs with the CDS system operating in silent mode.

**Main Outcomes and Measures:**

The primary outcome was the change in mean systolic blood pressure (SBP) between baseline and 180 days compared between groups. The primary analysis was a repeated measures linear mixed model, using SBP at baseline, 90 days, and 180 days in an intention-to-treat repeated measures model to account for missing data. Secondary outcomes included blood pressure (BP) control and outcomes such as percentage of patients who received an action that aligned with the CDS recommendations.

**Results:**

The study included 174 PCPs and 2026 patients (mean [SD] age, 75.3 [0.3] years; 1223 [60.4%] female; mean [SD] SBP at baseline, 154.0 [14.3] mm Hg), with 87 PCPs and 1029 patients randomized to the intervention and 87 PCPs and 997 patients randomized to usual care. Overall, 1714 patients (84.6%) were treated for hypertension at baseline. There were 1623 patients (80.1%) with an SBP measurement at 180 days. From the linear mixed model, there was a statistically significant difference in mean SBP change in the intervention group compared with the usual care group (change, −14.6 [95% CI, −13.1 to −16.0] mm Hg vs −11.7 [−10.2 to −13.1] mm Hg; *P* = .005). There was no difference in the percentage of patients who achieved BP control in the intervention group compared with the control group (50.4% [95% CI, 46.5% to 54.3%] vs 47.1% [95% CI, 43.3% to 51.0%]). More patients received an action aligned with the CDS recommendations in the intervention group than in the usual care group (49.9% [95% CI, 45.1% to 54.8%] vs 34.6% [95% CI, 29.8% to 39.4%]; *P* < .001).

**Conclusions and Relevance:**

These findings suggest that implementing this computerized CDS system could lead to improved management of uncontrolled hypertension and potentially improved clinical outcomes at the population level for patients with CKD.

**Trial Registration:**

ClinicalTrials.gov Identifier: NCT03679247

## Key Points

**Question:**

Is use of a computerized clinical decision support (CDS) intervention, including behavioral economic principles and human-centered design methods, associated with decreasing systolic blood pressure (SBP) for patients with chronic kidney disease and uncontrolled hypertension?

**Findings:**

In this randomized clinical trial including 184 randomized primary care practitioners and 2026 patients, patients of clinicians who used the CDS system had significantly greater SBP change at 180 days compared with the usual care group.

**Meaning:**

These findings suggest that implementing this computerized CDS system could lead to improved management of uncontrolled hypertension and potentially improved clinical outcomes for patients with chronic kidney disease at the population level.

## Teaser

This randomized clinical trial evaluates a computerized clinical decision support system for the management of uncontrolled hypertension in patients with chronic kidney disease.

## Introduction

Chronic kidney disease (CKD), defined by abnormal kidney function for greater than 3 months and quantified by an estimated glomerular filtration rate (eGFR) less than 60 mL/min/1.73 m^2^ or urine albumin to creatinine ratio (UACR) 30 mg/g or greater, affects 8% to 16% of the adult population globally.^1,2^ The prevalence of CKD has increased over time, which is partly attributed to the increase in CKD risk factors, including sociodemographic factors, genetic factors, and comorbidities, such as diabetes or hypertension.^3^ Hypertension affects 60% to 90% of people with CKD and is associated with worse kidney outcomes, as well as increased cardiovascular morbidity and mortality.^4^ Lowering blood pressure (BP) in patients with CKD has been associated with better cardiovascular and all-cause mortality and is a core goal in published guidelines for the treatment of this population of patients.^5^ Evidence-based management of hypertension in patients with CKD involves the use of renoprotective first-line agents, such as angiotensin-converting enzyme inhibitors (ACEs) and angiotensin receptor blockers (ARBs).^5,6,7,8^ Yet, despite national efforts to increase awareness of CKD, few patients with stage 3 to 4 CKD are aware of their disease; in addition, hypertension treatment in CKD remains suboptimal in primary care settings.^9,10,11^

Primary care practitioners (PCPs) play a critical role in reducing the burden of CKD by identifying patients with CKD and addressing risk factors, like hypertension and diabetes. Referral of patients to nephrology care is associated with cost and morbidity and mortality benefits, but access to ambulatory nephrology care does not match the need at a population level.^12^ Studies have shown that PCPs are not always aware of CKD management guidelines and face barriers to implementing them.^13,14^ Computerized clinical decision support (CDS) systems aim to close this gap by providing PCPs with person-specific information and timely evidence-based recommendations.^15^

Studies of CDS systems for CKD management have shown mixed results. Positive results have included decreased annualized loss of eGFR,^16^ increased rate of diagnosis,^17^ increased urine albumin testing,^17,18,19,20,21^ and increased referral to nephrologists.^21^ By contrast, other studies have shown no significant benefit of CDS for mitigating cardiovascular risk factors, including BP control.^16,17,19,20,22,23,24^ Unintended consequences of CDS impact its effectiveness.^25,26,27^ Behavioral economics has potential to increase effectiveness of CDS by addressing the psychological factors that impact decision-making.^28,29^ A behavioral economic nudge is a change in the choice architecture that may alter behavior in a predictable way while preserving options.^30,31,32^

The purpose of this randomized clinical trial was to examine whether PCP utilization of an intervention, including a computerized CDS system based on behavioral economic principles and human-centered design, leads to a decrease in systolic BP (SBP) compared with usual care.

## Methods

### Study Design and Setting

This randomized clinical trial was approved by the Human Subjects Institutional Review Board at Brigham and Women’s Hospital, with a waiver of informed consent for both PCPs and patients because of minimal risk to participants. PCPs were given the option to opt out of the study. The methods for this randomized clinical trial have been described in detail elsewhere,^33^ and the trial protocol and statistical analysis plan are provided in Supplement 1. This study followed the Consolidated Standards of Reporting Trials (CONSORT) reporting guideline. This study was conducted within the Brigham and Women’s Primary Care Practice-Based Research Network, which is nationally certified by the Agency for Healthcare Research and Quality. The network of 15 practices cares for 150 000 patients and includes hospital-based practices, ambulatory practices, and community health centers.

### Study Population

The target of the intervention was the PCP. All attending physicians, physician assistants, and nurse practitioners in our primary care network were assessed to determine whether they practiced as a PCP with a consistent panel of primary care patients.

Inclusion of patients for this clinical trial was based on real-time assessment of 2 inclusion criteria: CKD and uncontrolled hypertension. In other words, patients were not enrolled and did not provide informed consent, but instead were automatically included in the study if they met inclusion criteria. All patients aged 18 years or older who had a visit with a PCP at any of the intervention practices during the 2 years preceding the first visit during the study intervention period were eligible for inclusion. Once the study period began, each patient who had an office visit with a PCP and fulfilled criteria for CKD and uncontrolled hypertension were included in the study (eMethods in Supplement 2). The first inclusion criterion was stage 3 or stage 4 CKD, defined as 2 prior eGFR measures of 16 to 59 mL/min/1.73 m^2^, as calculated by non–race-corrected Chronic Kidney Disease Epidemiology Collaboration equation, within the previous 2 years separated by 90 days or 2 prior UACR measures greater than 30 mg/g within the previous 2 years separated by 90 days. The second inclusion criterion was uncontrolled hypertension, defined as at least 1 ambulatory SBP measure greater than 140 mm Hg within the 2 years preceding the visit at which the patient was assessed for inclusion in the study, as well as an elevated SBP measure at that baseline visit. Data collection ended on October 25, 2022.

### Randomization

**Figure 1.**
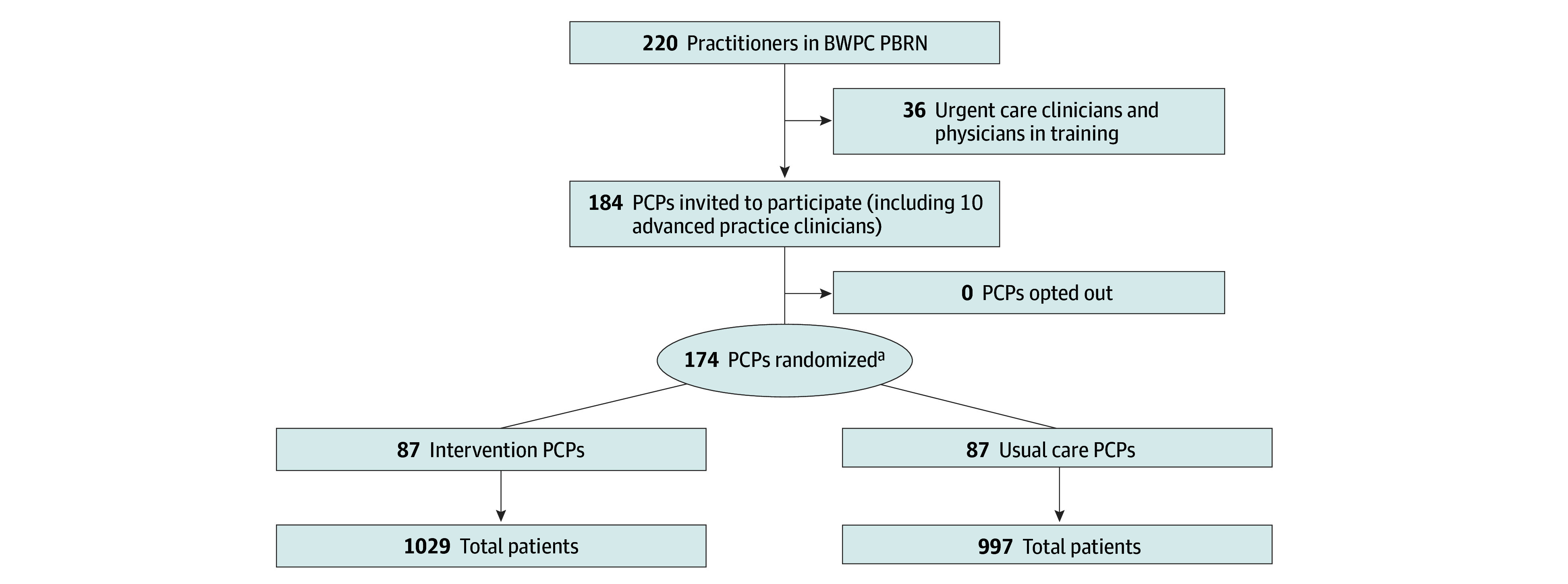
Participant Recruitment and Randomization Flowchart BWPC PBRN indicates Brigham and Women’s Primary Care Practice-Based Research Network; PCP, primary care practitioner. ^a^The number of PCPs randomized includes all individual physicians with their own panel of patients and 10 teams of a physician and an advanced practice clinician (ie, physician assistant or nurse practitioner) who share a panel.

This study used a matched-pair randomized design. One PCP in each pair was randomized to the intervention group and the other to the usual care group (Figure 1; eMethods in Supplement 2).

### Intervention

**Figure 2.**
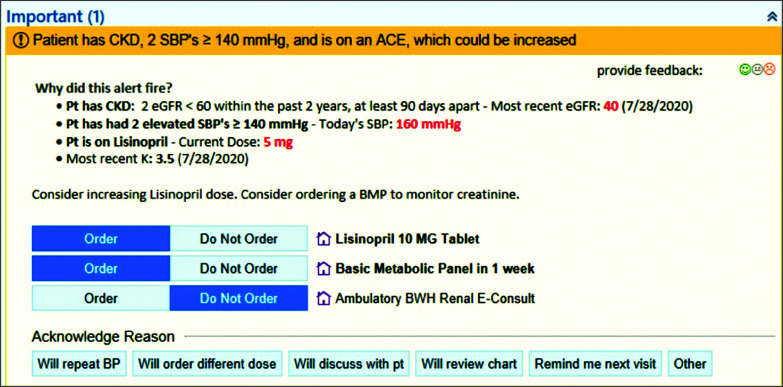
Example of Best Practice Advisory (BPA) for Computable Phenotype 2A Screenshot of BPA with explanation of trigger criteria, relevant clinical information, recommended actions, and required accountable justification (ie, Acknowledge Reason). Boxes represent items that initiate an action if selected, eg, the Order box for “Lisinopril, 10 mg, tablet” is selected, the medication will be ordered for the patient. By default, the Order boxes are selected for “Lisinopril 10 MG Tablet,” and “Basic Metabolic Panel in 1 week.” ACE indicates angiotensin-converting enzyme inhibitor; BMP, basic metabolic panel; BP, blood pressure; BWH, Brigham and Women's Hospital; CKD, chronic kidney disease; eGFR, estimate glomerular filtration rate; K, potassium; Pt, patient; SBP, systolic blood pressure.

One component of the intervention was the CDS system based in the electronic health record (EHR). The CDS was composed of a set of 5 Best Practice Advisories (BPAs), which we created and inserted into Epic Systems software. The iterative data analytic approach to developing the CDS is described in detail elsewhere.^34,35^ Briefly, we developed 5 computable phenotypes or algorithms to allow curation of disease subpopulations. For example, the third computable phenotype (2A) selected patients with both CKD and uncontrolled SBP who had lisinopril on the medication list at a dose lower than 40 mg. This computable phenotype triggered 1 of 5 BPAs, depending on the existing dose of lisinopril (2.5 mg, 5 mg, 10 mg, 20 mg, or 30 mg); for example, the BPA recommended 10 mg for a patient receiving 5 mg of lisinopril (Figure 2; eMethods in Supplement 2). Each BPA also included an order for basic metabolic panel. The orders for the recommended medication and basic metabolic panel were preselected to order, nudging the PCP to follow the recommendation. A third order was available for a nephrology electronic consult so that a PCP could choose to opt-in (as opposed to opt-out for medication and basic metabolic panel orders).

Another component of the intervention, developed using human-centered design principles, was the display of patient-specific data explaining why the CDS was triggered. Methods included contextual inquiry sessions and 2 rounds of usability testing, group design, and individual think-aloud sessions conducted virtually with PCPs.^25^

The intervention included several behavioral economic elements that were delivered internally and externally to the CDS. The internal elements were prechecked default orders that nudged PCPs toward recommended actions and a required accountable justification if the PCP did not place the orders that were recommended (Figure 2).^36,37^ The external behavioral economic element of the intervention was a precommitment email that was sent to intervention group PCPs asking them to pledge to follow recommendations about BP management or, if they chose not to do so, to enter an accountable justification.^38^ An email including a brief statement about CKD guidelines was sent to usual care group PCPs.^39^ While BPAs were only visible for clinicians in the intervention group, BPAs fired silently in the background for PCPs in the usual care group, allowing for the identification and follow-up of eligible patients who were not receiving the CDS intervention for the purpose of this analysis.

### Data Collection and Follow-Up

Patients were electronically identified and included in the study over a period of 365 days. Demographics, clinical characteristics, clinical comorbidities, and hypertension management data were collected from the EHR. Race and ethnicity were assessed through EHR data. Race was categorized as Asian, Black, White, or other (ie, any other entry, including free text, into the race field that did not indicate Asian, Black, or White), and ethnicity was categorized as Hispanic or not Hispanic. Race and ethnicity were included in analysis to obtain the demographic background of the study population. Due to the possibility that it would be clinically appropriate to defer action until kidney function was rechecked or an acute issue had resolved, the potential time frame for an effect of the intervention was expected to be approximately 6 months. Detailed description of data collection and follow-up are provided in the eMethods in Supplement 2.

### Outcomes

The primary outcome was the change in mean SBP between baseline and 180 days, compared between groups. The secondary BP outcome was the proportion of patients with controlled BP at 180 days (defined as BP <140/90 mm Hg). We examined orders for CDS-recommended actions in both groups during the encounter where the BPA was triggered (ie, the receipt of any ACE, ARB, or hydrochlorothiazide (HCTZ) order, the receipt of a basic metabolic panel order, or the receipt of a nephrology electronic consult). We also examined the initiation or dose titration of ACE, ARB, or HCTZ in subgroups of patients receiving each of the BPA phenotypes in both groups during the encounter when the BPA occurred. Finally, we had originally planned to report eGFR and UACR as secondary outcomes at 180 days, but given large amounts of missing data, we did not compare these outcomes across groups (amended in ClinicalTrials.gov: NCT03679247).

### Power

We assumed that 71% of included patients would have at least 1 follow-up visit during the follow-up period based on 2 previous studies of data from these primary care practices.^12,40^ We based our power calculation on an expected decrease in the mean of the final SBPs for patients in the intervention group of at least 3 mm Hg compared with the mean of the final SBPs in the usual care group, which is a clinically meaningful decrease at the population level. Using the mixed-model *z* test with a 2-sided type I error rate of 5%, we calculated that 497 evaluable patients per group and a mean of 6 patients per PCP would provide more than 80% power to detect a mean SBP decrease of 3 mm Hg in the intervention group compared with the usual care group (eMethods in Supplement 2).

### Statistical Analysis

Descriptive statistics for demographic and clinical characteristics between groups included percentages for categorical variables and means for continuous variables. Categorical demographic and clinical characteristics were compared across study groups using a Rao-Scott χ^2^ test, accounting for correlation within PCP panels by clustering by matched pairs of PCPs, and continuous demographic and clinical characteristics were compared using the Wilcoxon rank-sum test, accounting for correlation within PCP panels by clustering by matched pairs of PCPs.

For the primary outcome, we fit a repeated measures linear mixed model, in which the mean SBP at baseline, 90 days, and 180 days were modeled as a function of time (treating baseline, 3-month, and 6-month time points as class time covariates), treatment group, and time by treatment group interaction, using all outcome data from all time points for all patients in an intention-to-treat repeated measures model. In the hierarchical linear mixed model, to assess the independent association of the CDS with SBP, we included a random effect for PCP matched pair, a random effect for cluster (PCP) within pair, and an unstructured correlation matrix for the 3 repeated measures (baseline, 90-day, and 180-day SBP) within patients. We prespecified adjusted analyses, including the patient sociodemographic and clinical characteristics as covariates, in the adjusted mixed model if a characteristic showed significant difference between group (*P* < .05). Data were analyzed using SAS statistical software version 9.4 (SAS Institute) from October 26, 2022, to November 3, 2023.

## Results

### Study Participants

There were 174 PCPs randomized, and they cared for 2026 patients (mean [SD] age, 75.3 [0.3] years; 1223 [60.4%] female; mean [SD] SBP at baseline, 154.0 [14.3] mm Hg) who met the inclusion criteria from February 26, 2021, to February 25, 2022. The number of PCPs randomized includes all individual PCPs with their own panel of patients and 10 teams of a PCP and a midlevel clinician (physician assistant or nurse practitioner) who shared a panel. Four PCPs left the practices after randomization. One PCP opted out of receiving the BPAs (data included in intervention group for intention-to-treat analysis).

Stratified matched-pair randomization resulted in 1029 patients in the intervention group and 997 patients in the usual care group (Figure 1). Overall, there were 45 Asian patients (2.4%), 342 Black patients (17.9%), 1352 White patients (70.6%), and 176 patients (9.2%) identified as another race; 238 patients (12.6%) were Hispanic and 1659 patients (87.5%) were not Hispanic (Table 1). Clinical characteristics and documented clinical comorbidities did not differ between study groups (Table 1). Overall, 1714 patients (84.6%) were being treated for hypertension at baseline (Table 1). Hypertension regimens were similar in terms of number of agents prescribed (Table 1). Although most characteristics had similar distributions across the study groups, there were some differences (eg, sex).

**Table 1.** Patient Demographics and Clinical Characteristics at Baseline

Measurement variable	Patients, No./total No. (%)		
	Total (N = 2026)	Intervention (n = 1029)	Usual care (n = 997)
Age, mean (SD), y	75.3 (0.3)	74.2 (0.3)	75.42 (0.6)
Sex			
Male	803/2026 (39.6)	359/1029 (34.9)	444/997 (44.5)
Female	1223/2026 (60.4)	670/1029 (65.1)	553/997 (55.5)
Race			
Asian	45/1915 (2.4)	24/975 (2.5)	21/940 (2.2)
Black	342/1915 (17.9)	191/975 (19.6)	151/940 (16.1)
Other^a^	176/1915 (9.2)	79/975 (8.1)	97/940 (10.3)
White	1352/1915 (70.6)	681/975 (69.9)	671/940 (71.4)
Ethnicity			
Hispanic	238/1897 (12.6)	112/948 (11.8)	126/949 (13.3)
Not Hispanic	1659/1897 (87.5)	836/948 (88.2)	823/949 (86.7)
Highest level of education			
College	846/1892 (45.7)	434/958 (45.3)	430/934 (46.0)
High school	743/1892 (39.3)	380/958 (39.7)	363/934 (38.9)
<High school	211/1892 (11.2)	104/958 (10.9)	107/934 (11.5)
Other	74/1892 (3.9)	40/958 (4.2)	34/934 (3.6)
Preferred language			
English	1781/2020 (88.2)	903/1025 (88.1)	878/996 (88.2)
Spanish	173/2020 (8.6)	84/1025 (8.2)	89/996 (8.9)
Other^b^	66/2020 (3.3)	38/1025 (3.7)	28/996 (2.8)
Insurance type			
Medicaid	94/2026 (4.6)	45/1029 (4.4)	49/997 (4.9)
Medicare	1315/2026 (69.5)	677/1029 (65.8)	638/997 (64.0)
Private	617/2026 (30.5)	307/1029 (29.8)	310/997 (31.1)
Clinical characteristics			
SBP, mean (SD), mm Hg	154.0 (14.3)	154.3 (14.2)	153.7 (14.4)
Weight, mean (SD), lb	175.2 (44.3)	173.8 (43.5)	176.6 (45.0)
BMI, mean (SD)	28.96 (6.6)	28.94 (6.4)	28.97 (6.7)
Documented clinical comorbidities			
Type 2 diabetes	1320/1997 (66.1)	687/1011 (68.0)	633/986 (64.2)
Hypercholesteremia	1677/1997 (84.0)	842/1011 (83.3)	835/986 (84.7)
Underwent kidney transplant	15/2026 (0.7)	8/1029 (0.8)	7/997 (0.7)
Hypertension management at baseline^c^			
Treated for hypertension	1714/2026 (84.6)	872/1029 (84.7)	842/997 (84.5)
Antihypertensive medications in regimen, median (IQR)	2.0 (1.0-3.0)	2.0 (1.0-3.0)	2.0 (1.0-3.0)
Using RAAS inhibitor			
ACE	482/2026 (23.8)	244/1029 (23.7)	238/997 (23.9)
ARB	358/2026 (17.7)	191/1029 (18.6)	167/997 (16.8)
Direct renin inhibitor	0/2026	0/1029	0/997
ARNI	0/2026	0/1029	0/997
Diuretic medication			
Loop diuretic	328/2026 (16.2)	163/1029 (15.7)	165/997 (16.5)
Thiazide	293/2026 (14.5)	147/1029 (14.3)	148/997 (14.8)
MRA	7/2026 (0.35)	5/1029 (0.49)	2/997 (0.20)
Other diuretic	151/2026 (7.5)	96/1029 (9.2)	55/997 (5.6)
Calcium channel blocker	802/2026 (39.6)	405/1029 (39.4)	397/997 (39.8)
β-Blocker	861/2026 (42.5)	444/1029 (43.1)	417/997 (41.8)
Other antihypertensive	106/2026 (5.2)	61/1029 (5.9)	45/997 (4.5)

Abbreviations: ACE, angiotensin-converting enzyme inhibitor; ARB, angiotensin receptor blocker; ARNI, angiotensin receptor-neprilysin inhibitor; BMI, body mass index (calculated as weight in kilograms divided by height in meters squared); MRA, mineralocorticoid receptor antagonist; RAAS, renin-angiotensin-aldosterone system; SBP, systolic blood pressure.

^a^ Includes any other entry into the race field that did not indicate Asian, Black, or White, including free-text entry.

^b^ Includes any other entry into the language field that did not indicate English or Spanish, including free-text entry.

^c^ We defined the regimen of antihypertensive treatment at baseline as orders for antihypertensive medications over the previous 2 years that were still active at the time of the visit within the intervention period when the patient was included in the study. Antihypertensive medications are defined as any RAAS inhibitor, diuretic, calcium channel blocker, β-blocker, or other antihypertensive agents, such as vasodilators.

### Primary Outcome

There were 1623 patients (80.1%) with an SBP measurement at 180 days (815 patients in the intervention group; 808 patients in the usual care group). There was a significant difference in SBP change between groups, with a mean SBP change of −14.6 (95% CI, −13.1 to −16.0) mm Hg in the intervention group, compared with a mean SBP change of −11.7 (95% CI, −10.2 to −13.1) mm Hg in the usual care group (*P* = .005) (Table 2; eFigure in Supplement 2).

**Table 2.** SBP at Baseline and 180 Days, Change in Mean SBP from Baseline, and BP Control

Measurement variable	Intervention	Usual care	*P* value
Baseline SBP, mean (SD), mm Hg^a^	154.3 (14.2)	153.7 (14.4)	.54
SBP at 180 d, mean (SD), mm Hg^a^	139.5 (19.7)	142.1 (19.9)	.009
Change in SBP, % (95% CI), mm Hg^b^	−14.6 (−13.1 to −16.0)	−11.7 (−10.2 to −13.1)	.005
BP control, % (95% CI)^c^	50.4 (46.5 to 54.3)	47.1 (43.3 to 51.0)	.23

Abbreviations: BP, blood pressure; SBP, systolic blood pressure.

^a^ SBP at baseline is the observed mean SBP presented in Table 1. SBP at 180 days is a product of the model.

^b^ Mean change in SBP represents an adjusted estimate after controlling for baseline difference in sex, using a repeated measures model incorporating SBP measurements at baseline and 90 days, and after accounting for clustering by primary care practitioner matched pair, primary care practitioner, and patient.

^c^ By design, there were no patients with controlled BP at baseline. Percentage represents adjusted estimates after controlling for baseline difference in sex, using a repeated measures model incorporating BP measurements at 90 days, and after accounting for clustering by primary care practitioner matched pair, primary care practitioner, and patient. BP control was defined as BP less than 140/90 mm Hg.

### Secondary Outcomes

There was no significant difference in the percentage of patients who achieved BP control (defined as 140/90 mm Hg) at 180 days, with 50.4% (95% CI, 46.5% to 54.3%) of patients in the intervention group achieving control, compared with 47.1% (95% CI, 43.3% to 51.0%) of patients in the usual care group (Table 2). However, there was a statistically significant difference in the number of patients who received an action that aligned with the CDS recommendations in the intervention group, in which PCPs received the BPAs, compared with the usual care group, in which the BPAs occurred silently in the background (49.9% [95% CI, 45.1% to 54.8%] vs 34.6% [95% CI, 29.8% to 39.4%]; *P* < .001) (eTable 1 in Supplement 2). The percentage of patients who received an order for any ACE, ARB, or thiazide diuretic was higher in the intervention arm than in the usual care group (eg, 24.8% [95% CI, 21.2% to 28.3%] vs 10.0% [95% CI, 6.5% to 13.6%]; *P* < .001) (eTable 1 in Supplement 2). When we examined the initiation of ACE, ARB, or HCTZ in subgroups of patients who were not using these agents during the encounter in which the BPA occurred, we found that the percentage of patients who were initiated was higher in the intervention group than the usual care group (eTable 2 in Supplement 2). We also report the percentage of patients whose dose of ACE or ARB was increased, decreased, or refilled at the same dose (eTable 3 in Supplement 2). There was a large amount of missing data for eGFR and UACR at 180 days. eGFR was only ordered for 68% of patients. UACR was only ordered for 11% of patients.

## Discussion

In this multiclinic, clinician cluster randomized clinical trial, a CDS system incorporating behavioral economic principles and human-centered design methods resulted in a statistically significant reduction in SBP at the population level. In addition, the CDS increased prescriptions of ACE and ARB, which is an important step toward improving long-term clinical outcomes. PCPs in the intervention group also took other recommended actions (ie, basic metabolic panel, or nephrology electronic consult) more frequently than PCPs in the usual care group. However, despite the significant difference in SBP and these secondary outcomes, there was no significant difference between the intervention group and the usual care group in BP control: both groups increased the rate of control equally, from 0% to approximately 50%.

A 2020 meta-analysis of CDS across conditions and settings found small improvements in process measures, such as documentation, test ordering, and prescribing, but no improvement or even worsening of clinical outcomes, like SBP (median SBP for patients receiving an intervention increased by 1.0 mm Hg).^41^ Several studies of CDS in management of CKD have failed to show an effect on the primary outcome.^22,23^ One study found that a CKD CDS system successfully slowed annualized loss of eGFR.^16^ Another study of an EHR-based population health management system focused on slowing progression of CKD has finished enrollment, and results are pending.^42,43^ Previous studies have reported that an antihypertensive-focused CDS system for patients with CKD was successful in increasing UACR testing^17,20,21^ and nephrology referral.^21^ We believe that our study is the first to show a significant positive effect on SBP as a continuous outcome. However, we did not show a significant difference in BP control between study groups. Future studies of CDS for patients with CKD could follow the example of a study of a CDS system for patients with hypertension alone that, similarly to our study, synthesized data on the patient’s current antihypertensive regimen and made a recommendation to intensify treatment, but also incorporated physician and patient education in the group that successfully improved BP control.^44^ Improvements on our intervention could also include optimization of BPA firing within the workflow or CDS pathways to sequentially intervene on patients with CKD and uncontrolled BP.

The positive results of this study could be related to the behavioral economic elements of the intervention, which may be generalizable to other conditions. For example, our results align with those of a 2023 study conducted in a primary care setting in which the incorporation of behavioral economic components, like nudges, yielded statistically significant effects on statin prescribing.^45^ Other interventions have reduced prescription of high-risk medications to older adults and reduced inappropriate prescribing of antibiotics in upper respiratory tract infections.^37,38,46^

Similar CDS tools are likely to be effective in chronic conditions, like diabetes, because most diabetes is managed by PCPs who are motivated to provide evidence-based care but often experience clinical inertia during busy, time-pressured office visits. These tools could also help PCPs to prioritize tasks for people living with multiple chronic conditions.^47^

### Strengths and Limitations

The strengths of this study include the large sample size across a diverse primary care network, well-matched randomization, and low attrition rate of PCPs. Additionally, since the CDS system was developed using common EHR configuration tools, the CDS can be rapidly disseminated to improve care nationally. The increase in recommended orders in the intervention group indicates that the observed effect of the CDS may be mediated by an impact on PCP decision-making around antihypertensive management. Also, the recommended actions could be updated as guidelines change, for example, with the advent of sodium-glucose transport protein 2 (SGLT2) inhibitors in primary care management of CKD. Since the maximally tolerated dosing of ACE or ARB is preferred prior to initiation of an SGLT2 inhibitor, our findings will continue to be applicable.^48^ One common fear for both renin-angiotensin-aldosterone system inhibitors and SGLT2 inhibitors is the acute drop in eGFR, and this CDS tool includes guidance to address that concern.^49^

This study has several limitations. First, the absolute effect size was small. This size of effect was not clinically significant at the individual level, but previous studies suggest that a reduction in SBP of greater than 2 mm Hg is associated with clinically meaningful reductions in negative cardiovascular outcomes, such as coronary heart disease, stroke, and heart failure, at the population level.^50,51,52^ Second, although the 2 study groups were well matched in number of patients with CKD and patients’ baseline SBP, they were not equal in sex distribution or diastolic BP, likely due to the stratified, matched-pair randomization scheme. The difference in sex distribution could have been related to differences in PCP characteristics, such as PCP sex, but given that there is no evidence that PCP characteristics attenuate or intensify the effect of CDS tools, we did not stratify on PCP characteristics. The baseline differences in patient characteristics between study groups were accounted for by adjustment and clustering in the statistical analysis.

Third, the intended workflow is for the BPA to appear at the time that a PCP enters the examination room; this required the use of the first measurement of BP, which is often falsely elevated.^53^ However, even if the first BP measurement is inaccurate, it is the clinical data available to the PCP at the beginning of the visit and an elevated value should prompt further action, whether that be a repeated measurement or prescribing. The same concern about BP measurement error applies to the outcome assessment. The values may be subject to measurement error. If the measurement error is the same in both groups, which we expect due to randomization, the BP measurement error would not bias the estimates. Fourth, although there was a low attrition rate of PCPs, there were 4 PCPs who left the practices after randomization. Since each PCP was matched with another PCP for randomization, this could have caused imbalance between groups. We examined the number of patients seen by matched PCPs and found that 2 did not see any patients and 2 did see patients with CKD (9 patients for 1 PCP and 13 patients for the other PCP) and determined that the PCPs leaving the practice did not warrant a post hoc sensitivity analysis.

Fifth, due to selection of a population with elevated SBP, a large portion of the decrease in SBP from baseline to 180 days could be related to regression to the mean, but this effect would be equal in both study groups. Sixth, a limitation of the study design is that the effect in the intervention group could be due to the automated diagnosis of CKD and uncontrolled hypertension by the CDS system, rather than the other features of the CDS. A study design with a different control condition, in which the usual care group of PCPs are informed of these diagnoses, would isolate the effect of the CDS recommendations from the effect of the automated diagnosis. Seventh, the pragmatic study design was a likely contributor to many of these limitations and to missing data at 180 days, but testing interventions in clinical practice settings greatly improves generalizability of the findings over traditional randomized clinical trials.

## Conclusions

This randomized clinical trial found that patients whose PCPs were randomized to a CDS intervention based on behavioral economics principles and human-centered design methods experienced a statistically significant decrease in SBP at 180 days compared with the decrease in SBP of patients whose PCPs were randomized to the usual care group. The CDS intervention group had a modest improvement in SBP, but no difference in the proportion achieving adequate control. Further research is needed to understand the persistence of these findings and impact on CKD outcomes, such as cardiovascular disease and progression of kidney disease at the population level.

## Notes

Supplement 1.
Trial Protocol and Statistical Analysis Plan

Supplement 2.
eMethods.
**eTable 1.** PCP Orders During the Visit When the BPA Fired **eTable 2.** Change in PCP Behavior as Indicated by ACE, ARB, or HCTZ Medication Initiation During the Visit When the BPA Fired **eTable 3.** Change in PCP Behavior as Indicated by ACE/ARB Medication Titration During the Visit When the BPA Fired **eFigure.** Systolic Blood Pressure at Baseline and 180 Days

Supplement 3.
Data Sharing Statement
