# Supplementary material for: Clinical Decision Support for Hypertension Management in Chronic Kidney Disease: A Randomized Clinical Trial
Source: JAMA Intern Med. 2024 Mar 11;184(5):484–92. doi: 10.1001/jamainternmed.2023.8315 (PMC10928544; doi:10.1001/jamainternmed.2023.8315)
Supplement: Supplement 1. — Trial Protocol and Statistical Analysis Plan [file jamainternmed-e238315-s001.pdf]

SAP

Section 1: Administrative Information

- Protocol title and trial registration
  - 1a – Electronic Tools to Increase Recognition and Improve Primary Care Management for Hypertension in Chronic Kidney Disease
  - 1b – NCT03679247
- SAP version
  - 2 – Version 1 (7/21/23)
- Protocol version
  - 3 – 2018P000692
- SAP revisions
  - 4 – N/A
- Roles and responsibilities
  - 5 – Names, affiliations, and roles of SAP contributors
    - Ryan Dunk, Brigham and Women's Hospital, research assistant
    - John Kilgallon, Brigham and Women's Hospital, research assistant
    - Sarah Chen, Brigham and Women's Hospital, research assistant
- Signatures of:
  - 6a – Person writing the SAP – Ryan Dunk, BA; Sarah Chen, BS; John Kilgallon, BA
  - 6b – Senior statistician responsible – Stuart Lipsitz, ScD
  - 6c – Chief investigator/clinical lead – Lipika Samal, MD, MPH

Section 2: Introduction

- Background and rationale
  - 7 – Treating chronic kidney disease (CDK) in its early stages can prevent dialysis and reduce heart problems that go along with kidney disease. Computerized tools may help primary care doctors to diagnose the disease earlier and computer reminders may help doctors to prescribe the best treatments for hypertension (HTN), which will prevent dialysis and heart problems. In this project the investigators will create computer reminders that will then be tested in clinics to evaluate for improvement in treatment of high blood pressure in early chronic kidney disease.
- Objectives
  - 8 – To evaluate a multicomponent intervention for the management of uncontrolled hypertension in chronic kidney disease.
    - Hypothesis: The mean systolic blood pressure of the chronic kidney disease (CKD) population can be decreased by an intervention with three innovative features: 1) methods to synthesize electronic health record (EHR) data in order to identify under-diagnosed chronic conditions, 2) iterative improvement in clinical decision support (CDS) content through

human factors methods to maximize the "informativeness" of the CDS, and 3) the use of behavioral economic principles to create behavioral "nudges" internal and external to the CDS.

Section 3: Study Methods

- Trial design
  - 9 – A multicenter, pragmatic, controlled trial randomized at the clinician level will be conducted between February 2021 and February 2022 with follow-up through August 2022.
  - Intervention will include:
    - Clinical decision support (CDS) – composed of a set of five Epic Best Practices Advisories (BPAs). These BPAs were designed to utilize computable phenotypes (CPs) or algorithms that allow curation of disease subpopulations.
    - 5 CPs – each had its own CDS recommendation. The first CP will include patients with CKD and uncontrolled SBP for whom it was advised that an angiotensin converting enzyme inhibitor (ACEi) be prescribed. The second will include patients with CKD and uncontrolled SBP for whom an angiotensin receptor blocker (ARB) should be prescribed. The third CP will include patients who are on an ACEi but not at an optimal dose, while the fourth CP will include those who are on a suboptimal dose of an ARB and in each case a dose escalation will be recommended. The fifth CP will include patients who are maximized on an ACEi or ARB but are not on a diuretic.
    - User-centered design principles – these displays of patient specific data will explain why the CDS fired. User-centered design sessions will include contextual inquiry sessions and two rounds of usability testing (group design and individual think aloud sessions) on the CDS prototype. Additionally, hyperlinks to clinical guidelines supporting the CDS recommendation will be included in the final CDS.
- Randomization
  - 10 – This study will utilize a matched-pair randomized design. We will match pairs of PCPs with a similar number of patients and prior year mean blood pressure of patients. One PCP in each pair will be randomized to the intervention arm and the other to the usual care arm.
  - The pairing of PCPs induces a correlation of outcomes from patients in different PCPs within a pair, but the association is typically weaker.
  - PCPs will be stratified based on two factors (size of CKD panel and mean SBP). Within these strata, PCPs will be randomized to intervention and control arms in a 1:1 ratio. At the start of the study, each CKD patient seen by a participating PCP will be assigned to the same arm as their PCP. From there, the intervention will be in place for 12 months with data collection continuing for an additional 180 days.
- Sample size

    - 11 – 2026
- Framework
    - 12 – Superiority
- Statistical interim analyses and stopping guidance
    - No formal interim analyses were planned. The trial was deemed to be of low risk to the patient, but there was a DSMB that reviewed the trial every 12 months for adverse events (AEs).
    - 13a – N/A
    - 13b – N/A
    - 13c – N/A
- Timing of final analysis – 14
    - The trial will run from February 26, 2021, to February 25, 2022. Follow up will end on October 25, 2022.
    - Trial Notes:
        - Pilot Dates: 02/08/2021–02/25/2021
        - Trial Dates: 02/26/2021–02/25/2022
- Timing of outcome assessments – 15
    - Patients will be electronically identified and included in the study over the course of 12 months. Retrospective data indicates that 70% of patients will have a follow-up around 180 days. Outcomes assessment will occur at 180 days (+/- 60 days); and the measurement taken closest to 180 days will be used if multiple measurements were captured.

Section 4: Statistical Principles

- Confidence intervals and P values
    - 16 – $p<0.05$
    - 17 – Using a robust generalized estimating equations (GEE) repeated-measures linear mixed model z-test for continuous data with a two-sided type I error rate of 5%, we calculated that 497 evaluable patients per arm and an average of six patients per PCP would provide over 80% power to detect an average 3 mm Hg SBP decrease in the intervention arm. We assumed an intra-cluster (clinician) correlation coefficient of 0.1, as is commonly assumed in this type of cluster randomization study. Therefore, we have power to detect a 3 mm Hg decrease in mean of final SBPs in the intervention versus control arm.
    - 18 – Confidence intervals to be reported
        - 95% confidence interval
- Adherence and protocol deviations
    - 19a – To assess the reach of the CDS, statistics on the quantity and types of firings will be collected through enterprise data warehouse (EDW) and Epic queries. Concurrent manual review of Epic reports and chart review on CDS firing statistics will be conducted by team members to verify the automated monthly summaries. Analytic variables will include the percentage and types of clinicians in primary care who use the software, descriptions of excluded

clinicians, PCP review and/or response to pledge email, PCP interaction with the CDS, signing of orders or accountable justification documentation within the CDS, and whether the BPA fired appropriately during encounter.
  - 19b – Adherence to the intervention will be presented in the form of summary statistics on data from each PCPs interaction with the intervention.
  - 19c – Protocol deviations include PCPs no longer receiving the intervention due to opting out of the intervention midway through the trial, leaving the practice, or no longer seeing eligible patients.
  - 19d – All protocol deviations will be summarized in the final results paper.
- Analysis populations
  - 20 – Assigned interventions
    - Intervention arm – PCPs will receive guidance within electronic health record from clinical decision support system.
    - Control arm – PCPs in this arm will continue to provide usual care to patients.

Section 5: Trial Population

- Screening data – 21
  - N/A
- Eligibility – 22
  - Pragmatic clinical trial recruitment of patients will consist of real time assessment of two inclusion criteria: 1) CKD and 2) uncontrolled HTN. All patients over the age of 18 who have a visit with a PCP at one of the intervention practices during the 2 years preceding the visit date will be eligible for enrollment. Once the study period begins, each patient who has an office visit with a PCP and fulfills criteria for CKD and uncontrolled HTN will be enrolled in the study. The first inclusion criteria will be CKD, defined as two prior eGFRs 16-59 mL/min/1.73m^2 separated by 90 days, as calculated by non-race-corrected CKD-EPI, or two prior UACR>30mg/g separated by 90 days. The second inclusion criteria will be uncontrolled hypertension, defined as at least one SBP>140 mmHg within the 2 years preceding the enrollment visit, as well as SBP >140 mmHg at the enrollment visit. Patients with a most recent eGFR≤20 mL/min/1.73m^2 or two previous eGFRs within 2 years separated by at least 90 days ≤ 15 mL/min/1.73m^2 will be excluded.
- Recruitment – 23
  - We will report the total number of PCPs in the network, those included and invited to participate, those excluded (urgent care providers and physicians in training), those randomized, and those who opt out. Of those who are randomized, we will show the number randomized to the intervention group and the number randomized to the usual care group, as well as the number of patients recruited to each group.
- Withdrawal/follow-up

  - 24a & 24b – In the case of PCPs who leave the practice before the trial starts (but after randomization), we will examine the impact as well as the number of alerts received by their paired PCP in the other arm.
  - 24c – If the effect size is small, we will not make any adjustments and will refrain from running sensitivity analyses due. If the effect size is large, we will run sensitivity analyses to evaluate the overall repercussions of the dropout.
- Baseline patient characteristics – 25a and 25b
  - Baseline characteristics will include clinical characteristics of patients as well as any documented clinical comorbidities.
  - Clinical characteristics will include mean SBP at baseline, weight at baseline, and BMI at baseline.
  - Comorbidities documented at baseline will include type 2 diabetes, hypercholesteremia, and any kidney transplants.

Section 6: Analysis

Outcome definitions

- Primary Outcome Measure:
  - Change in mean systolic blood pressure (SBP) between baseline and 180 days compared across arms, a continuous variable
- Secondary Outcome Measures:
  - Overall BP control achieved at 180 days, a dichotomous variable (by study design, BP control at baseline was 0%)
  - Receipt of CDS-recommended actions by patients
    - any recommended action as well as any ACEi, ARB, or hydrochlorothiazide (HCTZ) ordered
  - 26a – specification of outcomes and timings.
    - The primary outcome will be the change in mean SBP between baseline and 180 days later, compared between arms. The secondary blood pressure outcome will be the fraction of patients with controlled BP at 180 days (defined as BP < 140/90 mmHg). Other secondary outcomes included the receipt of CDS-recommended actions by patients: receipt of any recommended action, the receipt of any ACEi, ARB, or hydrochlorothiazide (HCTZ) order, the receipt of a BMP order, and the receipt of a nephrology referral.
  - 26b – specific measurement and units
    - BP – mmHg
    - BMI – Kg/m 2
    - Weight – pounds
    - Other variables displayed as a percent (N/Total)
  - 26c – The primary outcome will be defined as the change from baseline to 180 days.
- Analysis methods

- The two arms will be compared using a robust GEE repeated-measures linear mixed model z-test for continuous data.
- Descriptive statistics for demographic and clinical characteristics between arms will include percentages for categorical variables and means or medians (with inter-quartile range) for continuous variables, where appropriate
- We will fit a robust generalized estimating equations (GEE) repeated-measures linear mixed model z-test for continuous data, in which the mean SBP at baseline, 90 and 180 days will be modelled as a function of time (treating baseline, 90 and 180 day time points as class time covariates), treatment arm, and time by treatment arm interaction, using all outcome data from all time points on all patients in an intention-to-treat repeated measures model. We will incorporate SBP measurements at 90 days to reduce the bias when estimating the 180 day mean SBP. Change in mean SBP from baseline to 180 days will be estimated using the repeated measures model. In the hierarchical linear mixed model, we will include a random effect for matched pair, a random effect for cluster (PCP) within pair, and an unstructured correlation matrix for the three repeated measures (baseline, 90 day and 180 day SBP) within patient.
- Categorical demographic and clinical characteristics will be compared across arms using a Rao-Scott chi-squared test (clustering by matched pairs of clinicians) and continuous demographic and clinical characteristics will be compared using the Wilcoxon rank-sum test (clustering by matched pairs of clinicians).
- We will consider including the patient characteristics as covariates in the mixed model if a characteristic shows significant difference between arms.
- 27d – The linear mixed model will protect against potential biases that could arise if patients in one arm are followed for longer periods and also will ensure that patients with better or worse SBP who have more follow-up will not disproportionately influence the assessment of the primary outcome.
- 27e – There will be no sensitivity analyses.
- 27f – There will be no subgroup analyses.

- Missing data – 28
    - The expectation–maximization algorithm will be used to estimate the linear mixed model. The expectation–maximization algorithm is equivalent to multiple imputation for a mixed model under an assumption of data missing at random.
- Additional analyses – 29
    - No additional statistical analyses will be required.
- Harms – 30
    - The investigators will establish an independent Data Safety Monitoring Board (DSMB), which will serve as an independent group to monitor participant safety, study burden and scientific validity of the clinical data.
    - The PI and co-investigators will review enrollment, adverse events and any recent literature that may be relevant to the research on a quarterly basis. Formal minutes with discussion points and remediation/actions plans (e.g. changes to protocol and consent documents) will be created, maintained, and relayed to the DSMB. The

    DSMB will convene in person or by teleconference once per year. At this meeting, the board will review any cases where systolic blood pressure was found to be below 110 mmHg, any reported adverse events, and deaths of enrolled patients.
  - In the event that the PI discovers that adverse events seem to occur more often in the intervention group, we will alert the IRB and DSMB and take further action as needed. Given our design, adverse events would be hypotension (SBP<110) and sequelae, such as falls.
- Statistical software – 31
  - Details of statistical packages to be used to carry out analyses
  - SAS Proc Mixed with a robust standard error will be used to carry out the main analysis, which will allow for clustering of patients within PCP's and PCP's within matched pairs. For the table one comparisons of baseline characteristics across arms, SAS Proc Surveyfreq will be used for categorical characteristics, and Proc Mixed for continuous characteristics, both using robust standard errors for clustering.
- References
  - 32a – N/A
  - 32b – N/A
  - 32c – N/A
  - 32d – N/A
