# Supplementary material for: Clinical Decision Support for Hypertension Management in Chronic Kidney Disease: A Randomized Clinical Trial
Source: JAMA Intern Med. 2024 Mar 11;184(5):484–92. doi: 10.1001/jamainternmed.2023.8315 (PMC10928544; doi:10.1001/jamainternmed.2023.8315)
Supplement: Supplement 2. — eMethods. eTable 1. PCP Orders During the Visit When the BPA Fired eTable 2. Change in PCP Behavior as Indicated by ACE, ARB, or HCTZ Medication Initiation During the Visit When the BPA Fired eTable 3. Change in PCP Behavior as Indicated by ACE/ARB Medication Titration During the Visit When the BPA Fired eFigure. Systolic Blood Pressure at Baseline and 180 Days [file jamainternmed-e238315-s002.pdf]

Supplemental Online Content

Samal L, Kilgallon JL, Lipsitz S, et al. Clinical decision support for hypertension management in chronic kidney disease: a randomized clinical trial. *JAMA Intern Med*. Published online March 4, 2024. doi:10.1001/jamainternmed.2023.8315

eMethods.
eTable 1. PCP Orders During the Visit When the BPA Fired
eTable 2. Change in PCP Behavior as Indicated by ACE, ARB, or HCTZ Medication Initiation During the Visit When the BPA Fired
eTable 3. Change in PCP Behavior as Indicated by ACE/ARB Medication Titration During the Visit When the BPA Fired
eFigure. Systolic Blood Pressure at Baseline and 180 Days

This supplemental material has been provided by the authors to give readers additional information about their work.

© 2024 Samal L et al. *JAMA Internal Medicine.*

eMethods

Detailed Inclusion Criteria

The first inclusion criterion was stage 3 or stage 4 CKD, defined as two prior eGFRs 16-59 mL/min/1.73m^2 as calculated by non-race-corrected CKD-EPI within the previous 2 years separated by 90 days, or two prior UACR>30mg/g within the previous 2 years separated by 90 days.[1] Inpatient eGFRs were included. We aligned our criteria with the KDIGO guidelines which do not differentiate between an eGFR decrease during AKI versus a lasting change in eGFR. One rationale for this approach is that AKI is correlated with the same poor outcomes as CKD – major adverse kidney events and cardiovascular events, particularly in the context of lowering BP. The second inclusion criterion was uncontrolled hypertension, defined as at least one systolic blood pressure (SBP)>140 mmHg within the 2 years preceding the first visit within the intervention period when the patient was included in the study, as well as SBP>140 mmHg at the first visit within the intervention period when the patient was included in the study. Only ambulatory blood pressure measurements were used. If the BP was repeated, the first measurement was included in our analysis since that measurement was reflected in the CDS alert. Patients with stage 1 or 2 CKD as well as stage 5 or end-stage kidney disease (defined as two previous eGFRs ≤ 15 mL/min/1.73m^2 within 2 years separated by at least 90 days) were excluded. Post-transplant patients with stage 3 or stage 4 CKD by the eGFR or UACR definition were included. CKD patients may or may not have been regularly seeing a nephrologist.

Randomization

This study utilized a matched-pair randomized design. Prior to inclusion of patients, we matched pairs of PCPs with a similar number of patients and prior year mean SBP of CKD patients (randomization conducted by SL). One PCP in each pair was randomized to the intervention arm and the other to the usual care arm. In cases where a panel of patients was co-managed by a PCP and a PA or NP, the physician and PA or NP were considered a single PCP for randomization and these teams were treated the same way as the rest of the PCPs in the randomization process.

Intervention

One component of the intervention was electronic health record (EHR)-based CDS, composed of a set of five Epic Best Practice Advisories (BPAs). BPAs are a type of CDS commonly used in Epic to provide targeted, patient-specific clinical guidance at the point of care. We developed five computable phenotypes or algorithms to allow curation of disease subpopulations, described in detail elsewhere.[2,3] The first computable phenotype (1A) selected patients with both CKD and uncontrolled SBP (as defined in the *Study Population* section) who did not have an ACE or ARB on the medication list. The BPA presented a one-click order for a 5 mg dose of the ACE lisinopril and a basic metabolic panel to be collected in seven days. The second computable phenotype (1B) selected patients with both CKD and uncontrolled SBP, who did not have an ACE or ARB on the medication list and had an allergy to ACE documented on the allergy list. The BPA presented a one-click order for a 25 mg dose of the ARB Losartan and a basic metabolic panel to be collected in seven days. The third computable phenotype (2A) selected patients with both CKD and uncontrolled SBP, who had lisinopril on the medication list at a dose lower than 40 mg. This computable phenotype triggered one of five BPAs depending on the existing dose of lisinopril (2.5 mg, 5 mg, 10 mg, 20 mg, or 30 mg); for example, the BPA recommended 20 mg for a patient on 10 mg of lisinopril. The fourth computable phenotype (2B) selected patients with both CKD and uncontrolled SBP, who had losartan on the medication list at a dose lower than 100 mg (25 mg or 50 mg). This computable phenotype triggered one of two BPAs depending on the existing dose of losartan; for example, the BPA recommended 50 mg for a patient on 25 mg of losartan. The BPAs for the third and fourth computable phenotype also included an order for a basic metabolic panel to be collected in seven days. The fifth computable phenotype (3A) selected patients with both CKD and uncontrolled SBP with either lisinopril 40mg or losartan 100 mg on the medication list and no diuretic on the medication list. The BPA presented a one-click order for hydrochlorothiazide (HCTZ) 12.5 mg and an order for a basic metabolic panel to be collected in seven days. There was a third order available for a nephrology electronic consult for which a PCP could choose to “opt-in” as opposed to the “opt-out” for medication and basic metabolic panel orders.

© 2024 Samal L et al. *JAMA Internal Medicine.*

Another component of the intervention, developed using human-centered design principles, was the display of patient-specific data explaining why the CDS was triggered. A description of the human-centered design process has been described in detail previously, but briefly, methods included contextual inquiry sessions and two rounds of usability testing, group design and individual think aloud sessions, conducted virtually with PCPs.[4] An example of the results of the human-centered design sessions was the addition of a statement, “An increase in Cr [serum creatinine] of up to 30% is acceptable after starting ACE” to the first BPA due to PCPs’ concern about this phenomenon. Due to this feedback, the BPA also included an order for a basic metabolic panel to be completed seven days after the prescription of the ACE. Additionally, hyperlinks to clinical guidelines supporting the CDS recommendation and the option to submit an electronic consult to nephrology were included in the final CDS.[5,6]

The intervention included several behavioral economic elements that were delivered internally and externally to the CDS. The internal elements were 1) pre-checked default orders that “nudged” PCPs toward recommended actions and 2) a required “accountable justification” if the PCP did not place the orders that were recommended (Figure 2).[7,8] In cases where a PCP considered taking action at a future time, one option was to click “Remind Me at Next Visit” which would “snooze” the BPA until a subsequent visit. The external behavioral economic element was a pre-commitment email that was sent to intervention arm PCPs asking them to pledge to follow recommendations about blood pressure management or, if they chose not to do so, to enter an accountable justification.[9] An email including a brief statement about CKD guidelines was sent to usual care arm PCPs.[6] The control condition for patients in the usual care arm was that they received routine clinical care at the PCP’s discretion throughout the study period. PCPs in the usual care arm did not see any BPAs and were not notified that the individual patients in the usual care arm had CKD and uncontrolled HTN. While BPAs were only visible for clinicians in the intervention arm, BPAs fired “silently” in the background for PCPs in the usual care arm, allowing for the identification and follow-up of eligible patients who were not receiving the CDS intervention for the purpose of this analysis.

Power

We assumed that 71% of the included patients would have at least one follow-up visit during the follow-up period based on two previous studies of data from these primary care practices.[10,11] Combining our findings from the two studies, we expected 42% of primary care patients with CKD to meet the inclusion criteria for uncontrolled HTN. We based our power calculation on an expected decrease in the mean of the final SBPs for patients in the intervention arm of at least 3 mmHg as compared to the mean of the final SBPs in the usual care arm, which is a clinically meaningful decrease at the population level.

The two arms were compared using a robust repeated-measures linear mixed-model z-test for continuous data; this approach does not assume normality of the outcome, and accounts for a possible cluster effect of patients between matched pairs of PCPS and within PCP.[11,12] Using the mixed-model z-test with a 2-sided type I error rate of 5%, we calculated that 497 evaluable patients per arm and an average of 6 patients per PCP would provide over 80% power to detect an average 3 mmHg SBP decrease in the intervention arm as compared to the usual care arm. We assumed that the intraclass correlation coefficient (ICC) for patients with the same PCP within a pair is approximately 0.1 as is commonly assumed in this type of cluster randomization study[13]; the ICC for patients from different PCPs in the same matched pair was assumed to be half (approximately 0.05) of the ICC for patients from the same PCP.

Statistical Analysis

Descriptive statistics for demographic and clinical characteristics between arms included percentages for categorical variables and means for continuous variables. Categorical demographic and clinical characteristics were compared across study arms using a Rao-Scott chi-squared test, accounting for correlation within PCP panels by clustering by matched pairs of PCPs, and continuous demographic and clinical characteristics were compared using the Wilcoxon rank-sum test, accounting for correlation within PCP panels by clustering by matched pairs of PCPs.

For the primary outcome, we fit a repeated measures linear mixed model, in which the mean SBP at baseline, 90 days, and 180 days were modeled as a function of time (treating baseline, 3-month and 6-month time points as class time covariates), treatment arm, and time by treatment arm interaction, using all outcome

© 2024 Samal L et al. *JAMA Internal Medicine.*

data from all time points on all patients in an intention-to-treat repeated measures model. In the hierarchical linear mixed model, to assess the independent association of the CDS with SBP, we included a random effect for PCP matched pair, a random effect for cluster (PCP) within pair, and an unstructured correlation matrix for the three repeated measures (baseline, 90-day and 180-day SBP) within patient. We prespecified adjusted analyses including the patient sociodemographic and clinical characteristics as covariates in the adjusted mixed model if a characteristic showed significant difference between arms ($p<0.05$). The expectation–maximization algorithm was used to estimate the linear mixed model,[14] and is equivalent to multiple imputation for a mixed model under an assumption of data missing at random.[15,16] From this repeated measures linear mixed model, we estimated the mean change in SBP from baseline to 180 days in the two arms. We incorporated SBP measurements at 90 days to reduce the bias when estimating the 180-day mean SBP. Although length of follow-up did not differ between arms, the linear mixed model protected against potential biases that could arise if patients in one arm were followed for longer periods than patients in the other arm and also ensured that patients with better or worse SBP at baseline or at 90 days who had follow-up at 180 days did not disproportionately influence the assessment of the primary outcome.

© 2024 Samal L et al. *JAMA Internal Medicine.*

eTable 1. PCP orders during the visit when the BPA fired.

Measurement Variable	Total N=2026	Intervention N=1029	Usual Care N=997	p-value
Receipt of Any Action that Aligned with CDS Recommendations, % (95% CI) [a, b]	42.3 (38.60-45.93)	49.9 (45.08-54.79)	34.6 (29.77-39.43)	**p <.0001**
Any ACE, ARB, or thiazide diuretic ordered, % (95% CI)[a]	17.4 (14.86-19.94)	24.8 (21.18-28.34)	10.0 (6.48-13.60)	**p<0.001**

[a] Percentage represents adjusted estimates after controlling for baseline difference in sex and after accounting for clustering by PCP matched pair and PCP.

[b] Actions aligned with CDS Recommendations included orders for ACE, ARB or thiazide diuretic, basic metabolic panel orders, and nephrology electronic consult orders

© 2024 Samal L et al. *JAMA Internal Medicine.*

eTable 2. Change in PCP behavior as indicated by ACE, ARB, or HCTZ medication initiation during the visit when the BPA fired.

**BPA Phenotype**	PCP Initiated Medication		**P value ***
	**Intervention**	**Usual Care**	
1A, Initiate Lisinopril 5 mg, N/Total patients who received the 1A BPA phenotype (%)	65/443 (14.67)	8/479 (1.67)	**<0.0001**
1B, Initiate Losartan 50 mg, N/ Total patients who received the 1B BPA phenotype (%)	25/139 (17.98)	5/103 (4.85)	**0.006**
3A, Initiate Hydrochlorothiazide, 12.5 mg, if patient is on maximal ACE or ARB, N/ Total patients who received the 3A BPA phenotype (%)	11/100 (11.00)	2/94 (2.13)	**0.014**

*P values accounting for clustering by PCP matched pair and PCP.

© 2024 Samal L et al. *JAMA Internal Medicine.*

eTable 3. Change in PCP behavior as indicated by ACE/ARB medication titration during the visit when the BPA fired.

BPA Phenotype	Dose Ordered by PCP							
	Increase to BPA Recommended Dose [a]		Increase [b]		Stable		Decrease	
	**Intervention**	**Usual Care**	**Intervention**	**Usual Care**	**Intervention**	**Usual Care**	**Intervention**	**Usual Care**
2A, Increase Lisinopril prescription from 2.5 mg to 5mg, N/Total (%)	1/1 (100)	2/3 (66.66)	1/1 (100)	2/3 (66.66)	0/1 (0)	1/3 (33.33)	0/1 (0)	0/3 (0)
2A, Increase Lisinopril prescription from 5 mg to 10 mg, N/Total (%)	4/8 (50.00)	2/7 (28.57)	4/8 (50.00)	2/7 (28.57)	4/8 (50.00)	4/7 (57.14)	0/8 (0)	1/7 (14.29)
2A, Increase Lisinopril prescription from 10 mg to 20 mg, N/Total (%)	4/17 (23.53)	3/7 (42.86)	4/17 (23.53)	3/7 (42.86)	13/17 (76.47)	3/7 (42.86)	0/17 (0)	1/7 (14.29)
2A, Increase Lisinopril prescription from 20 mg to 40 mg, N/Total (%)	7/25 (28.00)	1/10 (10.00)	9/25 (36.00)	1/10 (10.00)	16/25 (64.00)	8/10 (80.00)	0/25 (0)	1/10 (10.00)
2A, Increase Lisinopril prescription from 30 mg to 40 mg, N/Total (%)	3/5 (60.00)	2/5 (40.00)	3/5 (60.00)	2/5 (40.00)	2/5 (40.00)	3/5 (60.00)	0/5 (0)	0/5 (0)

© 2024 Samal L et al. *JAMA Internal Medicine.*

2B, Increase Losartan prescription from 25 mg to 50 mg, N/Total (%)	10/15 (66.66)	2/5 (40.00)	10/15 (66.66)	3/5 (60.00)	5/15 (33.33)	2/5 (40.00)	0/15 (0)	0/5 (0)
2B, Increase Losartan prescription from 50 mg to 100 mg, N/Total (%)	10/30 (33.33)	3/16 (18.75)	10/30 (33.33)	3/16 (18.75)	17/30 (56.66)	11/16 (68.75)	3/30 (10.00)	2/16 (12.50)

[a] Percentages represent PCP orders that increased an ACE/ARB medication dose exactly as recommended by this BPA.

[b] Percentages represent PCP orders that increased an ACE/ARB medication dose exactly as recommended by this BPA in addition to PCP orders that increased ACE/ARB medication dose, but to a dose that was not recommended by this BPA.

© 2024 Samal L et al. *JAMA Internal Medicine.*

eFigure. Mean Systolic Blood Pressure at Baseline and 180 Days

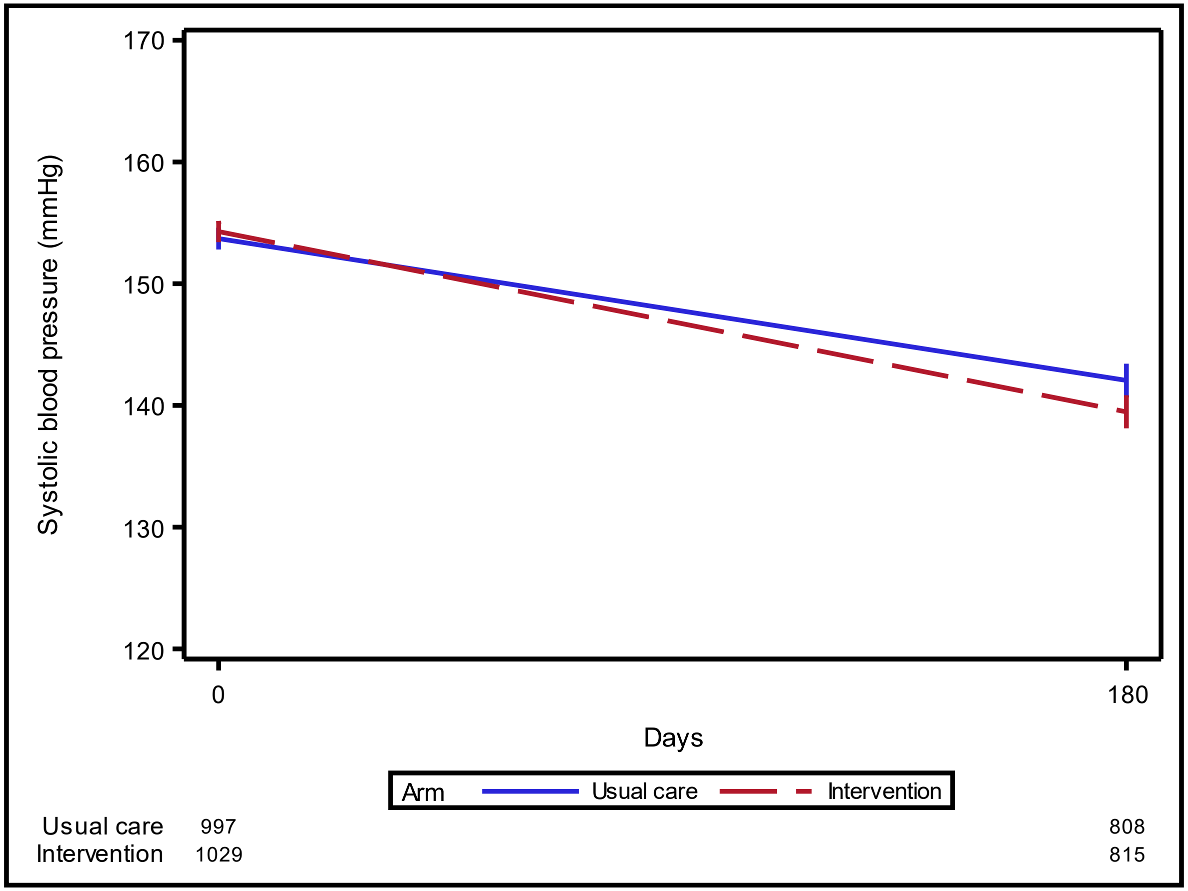

Mean systolic blood pressure (SBP) at baseline and 180 days in both intervention and usual care arms. Error bars represent the standard deviation from the mean. The total number of participants with SBP values available in the intervention arm is 1029 at baseline and 815 at 180 days. The total number of participants with SBP values available in the usual care arm is 997 at baseline and 808 at 180 days.

© 2024 Samal L et al. *JAMA Internal Medicine.*

References

1. Delgado C, Baweja M, Crews DC, et al. A Unifying Approach for GFR Estimation: Recommendations of the NKF-ASN Task Force on Reassessing the Inclusion of Race in Diagnosing Kidney Disease. *Journal of the American Society of Nephrology*. 2021;32(12):2994-3015. doi:10.1681/asn.2021070988
2. Samal L, Wu E, Aaron S, et al. Refining Clinical Phenotypes to Improve Clinical Decision Support and Reduce Alert Fatigue: A Feasibility Study. *Appl Clin Inform*. May 2023;14(3):528-537. doi:10.1055/s-0043-1768994
3. Gannon MP, Wu E, McMahon GM, et al. Uncontrolled blood pressure and treatment of hypertension in older chronic kidney disease patients. *J Am Geriatr Soc*. Oct 2021;69(10):2985-2987. doi:10.1111/jgs.17304
4. Garabedian PM, Gannon MP, Aaron S, Wu E, Burns Z, Samal L. Human-centered design of clinical decision support for management of hypertension with chronic kidney disease. *BMC Med Inform Decis Mak*. Aug 13 2022;22(1):217. doi:10.1186/s12911-022-01962-y
5. Sinha AD, Agarwal R. Clinical Pharmacology of Antihypertensive Therapy for the Treatment of Hypertension in CKD. *Clin J Am Soc Nephrol*. May 7 2019;14(5):757-764. doi:10.2215/CJN.04330418
6. Levey AS. K/DOQI clinical practice guidelines on hypertension and antihypertensive agents in chronic kidney disease. American Journal of Kidney Diseases. https://kidneyfoundation.cachefly.net/professionals/KDOQI/guidelines_bp/guide_11.htm
7. Patel MS, Day S, Small DS, et al. Using default options within the electronic health record to increase the prescribing of generic-equivalent medications: a quasi-experimental study. *Ann Intern Med*. Nov 18 2014;161(10 Suppl):S44-52. doi:10.7326/m13-3001
8. Meeker D, Linder JA, Fox CR, Friedberg MW, Persell, S.D., Goldstein, N.J., Knight, T.K., Hay, J.W., Doctor, J.N. Effect of behavioral interventions on inappropriate antibiotic prescribing among primary care practices: A randomized clinical trial. *JAMA*. Feb 9, 2016 2016;315(6):562-70.
9. Lauffenburger JC, Isaac T, Trippa L, et al. Rationale and design of the Novel Uses of adaptive Designs to Guide provider Engagement in Electronic Health Records (NUDGE-EHR) pragmatic adaptive randomized trial: a trial protocol. *Implement Sci*. Jan 7 2021;16(1):9. doi:10.1186/s13012-020-01078-9
10. Samal L, Wright A, Waikar SS, Linder JA. Nephrology co-management versus primary care solo management for early chronic kidney disease: a retrospective cross-sectional analysis. *BMC Nephrol*. Oct 12 2015;16:162. doi:10.1186/s12882-015-0154-x
11. Singh K, Waikar SS, Samal L. Evaluating the feasibility of the KDIGO CKD referral recommendations. *BMC Nephrology*. 2017/07/07 2017;18(1):223. doi:10.1186/s12882-017-0646-y
12. Jaspers MW, Steen T, van den Bos C, Geenen M. The think aloud method: a guide to user interface design. *Int J Med Inform*. Nov 2004;73(11-12):781-95. doi:10.1016/j.ijmedinf.2004.08.003

© 2024 Samal L et al. *JAMA Internal Medicine.*

13. Rieckert A, Teichmann AL, Drewelow E, et al. Reduction of inappropriate medication in older populations by electronic decision support (the PRIMA-eDS project): a survey of general practitioners' experiences. *J Am Med Inform Assoc*. Nov 1 2019;26(11):1323-1332. doi:10.1093/jamia/ocz104
14. Chen J, Zhang D, Davidian M. A Monte Carlo EM algorithm for generalized linear mixed models with flexible random effects distribution. *Biostatistics*. Sep 2002;3(3):347-60. doi:10.1093/biostatistics/3.3.347
15. Ibrahim J, Chen M-H, Lipsitz S, Herring A. Missing-Data Methods for Generalized Linear Models: A Comparative Review. *Journal of the American Statistical Association*. 02/01 2005;100:332-346. doi:10.2307/27590542
16. Lin TH. A comparison of multiple imputation with EM algorithm and MCMC method for quality of life missing data. *Quality & Quantity*. 2010/02/01 2010;44(2):277-287. doi:10.1007/s11135-008-9196-5

© 2024 Samal L et al. *JAMA Internal Medicine.*
