# Supplementary material for: Clinical Decision Support for Hypertension Management in Chronic Kidney Disease: A Randomized Clinical Trial
Source: JAMA Intern Med. 2024 Mar 11;184(5):484–92. doi: 10.1001/jamainternmed.2023.8315 (PMC10928544; doi:10.1001/jamainternmed.2023.8315)
Supplement: Supplement 3. — Data Sharing Statement [file jamainternmed-e238315-s003.pdf]

Data Sharing Statement

Samal. Clinical Decision Support for Hypertension Management in Chronic Kidney Disease. *JAMA Intern Med*. Published March 11, 2024. doi:10.1001/jamainternmed.2023.8315

Data
Data available: No

Additional Information
Explanation for why data not available: To protect patient privacy and confidentiality, we will not be sharing individual level de-identified data. Aggregate data sets will be made available upon reasonable request.
